# Investigation of precursor lesions of polypoidal choroidal vasculopathy using contralateral eye findings

**DOI:** 10.1007/s00417-016-3452-5

**Published:** 2016-09-05

**Authors:** Se Woong Kang, Hoyoung Lee, Kunho Bae, Joo Young Shin, Sang Jin Kim, Jong Min Kim, for the Korean Age-related Maculopathy Study (KARMS) Group, Se Woong Kang, Sang Jin Kim, Suk Ho Byeon, Kyu Hyung Park, Jae Pil Shin, Seung Young Yu, Jaeryung Oh, Young-Joon Jo, Hyun Woong Kim

**Affiliations:** 1 Department of Ophthalmology, Samsung Medical Center, Sungkyunkwan University School of Medicine, #81 Irwon-ro, Gangnam-gu, Seoul 06351 Republic of Korea; 2 Department of Ophthalmology, Seoul National University College of Medicine, Seoul National University Hospital Healthcare System Gangnam Center, Seoul, Korea

**Keywords:** Age-related macular degeneration, Drusen-like deposit, Pigmentary change, Polypoidal choroidal vasculopathy

## Abstract

**Purpose:**

The purpose was to investigate precursor lesions of polypoidal choroidal vasculopathy (PCV).

**Methods:**

This cross-sectional study involved 276 unaffected contralateral eyes from unilateral PCV patients (Group 1), unilateral typical exudative age-related macular degeneration (AMD) patients (Group 2), and unilateral epiretinal membrane patients (Group 3) as age-matched controls. Grayish-yellow sub-retinal or sub-retinal-pigment-epithelial deposits larger than 63 μm in size with irregular but discrete margins were defined as drusen-like deposits (DLDs). The frequencies of DLDs, drusen, and pigmentary changes in each group were compared.

**Results:**

DLDs larger than 125 μm in size were found more frequently in Group 1 (19.5 %) than in Groups 2 (3.4 %) and 3 (3.2 %) (*p <* 0.001). Soft drusen were discovered more frequently in Group 2 eyes than in Groups 1 and 3 (*p* < 0.001). Pigmentary changes were found more frequently in Groups 1 and 2 compared to Group 3. Compared with the other groups, Group 1 manifested a higher frequency of choroidal vascular hyperpermeability (*p* < 0.005) and thicker choroid (*p* < 0.001).

**Conclusions:**

The precursor lesions of PCV are different from those of exudative AMD. DLDs larger than 125 μm and pigmentary changes may be early preclinical markers of PCV. Long-term longitudinal studies are warranted for validation.

## Introduction

Soft drusen and sub-retinal pigmentary changes in the macular area are well-known clinical markers of early age-related macular degeneration (AMD). Previous studies such as the Age-Related Eye Disease Study suggest that the number and size of soft drusen and pigmentary changes are associated with the risk of exudative AMD [1–3], and AMD grading systems based on these findings are currently used as clinical markers.

There is a preponderance of polypoidal choroidal vasculopathy (PCV) among neovascular AMD in Asian patients. PCV accounts for 54.7 % of the neovascular AMD in a Japanese study [4]. Recent study has also reported the proportion of PCV among neovascular AMDs to be different according to geographic location, 9 % in Paris, and 48 % in Kyoto [5]. Because PCV and typical exudative AMD share certain common environmental risk factors, genetic determinants, and a similar presentation of serosanguineous maculopathy, PCV has been categorized as a subtype of AMD [6]. However, PCV shows distinct characteristics from other neovascular AMD in that it is exclusively accompanied by multifocal choroidal hyperfluorescence and venous engorgement on indocyanine green angiography (ICGA) [7–10]. Also, subfoveal choroidal thicknesses in eyes with PCV are much thicker than the eyes with typical exudative AMD [11, 12]. Furthermore, PCV is characterized by slower progression, better visual prognosis despite more chronic persistence when compared with typical exudative AMD [13, 14]. Therefore, there has been some controversy whether PCV should be categorized as a subtype of AMD.

The precursor lesion of active PCV is not known. There is no definite evidence that soft drusen or sub-retinal pigmentary changes are early lesions of PCV. Previous studies have reported different characteristics between early lesions of PCV and typical exudative AMD, such as that soft drusen, are found less frequently, while pigmentary abnormalities only are found more frequently in PCV than in AMD [6, 15, 16]. Ueta et al., in their fellow eye study of PCV, observed that retinal pigmentary atrophy was a prevailing finding before the manifestation of active PCV [15]. However, the significance of putative risk factors of PCV has not been clearly addressed. Identifying the early lesion of PCV, which is highly prevalent in Asians, might contribute to understanding the pathophysiology of PCV and aid in the development of preventive measures. Studying the contralateral eye of unilaterally active PCV patients and comparing them with an age-matched control group of eyes might be one of the measures to investigate the precursor lesions, because the probability of a PCV patient developing PCV in the contralateral eye is higher than the probability of normal age-matched person developing PCV [4, 7, 15].

This study was conducted to investigate the precursor lesions of PCV by studying the uninvolved contralateral eye of active unilateral PCV patients compared with age-matched control eyes and the uninvolved contralateral eye of patients with unilateral typical exudative AMD.

## Materials and methods

The study protocol was approved by the Institutional Review Board of Samsung Medical Center (IRB No. 2015-05-086), and conformed to the Declaration of Helsinki. Patient records were anonymized and de-identified prior to analysis.

### Study participants

Patients with unilateral PCV who visited one of seven referral hospitals in Korea from July 2011 to July 2014 were included in this study. The patients with a unilateral typical exudative AMD or a unilateral epiretinal membrane (ERM), who visited Samsung Medical Center during the same period, were also included for comparison. The patients were divided into three groups, and the uninvolved contralateral eyes were analyzed. Group 1 consisted of the contralateral eyes of unilateral PCV patients, Group 2 consisted of the contralateral eyes of unilateral typical exudative AMD patients, and Group 3 consisted of the contralateral eyes of unilateral ERM patients as age-matched controls (Datasets are included in the S1 appendix). Retinal specialists at each center reviewed fundus photography, spectral-domain optical coherence tomography (OCT), fluorescein angiography, and confocal indocyanine green angiography (ICGA) images of PCV and typical exudative AMD patients, and the images were reviewed by two separate retinal specialists (GEC, HYL) in order to confirm the diagnosis. Patients that had a concordant diagnosis were included in the study. When the diagnosis was different among the reviewers, another retina specialist (SWK) reviewed the images and discussed the findings until a final diagnosis was reached and the patient group was determined.

Inclusion criteria were as follows: 50 years of age or older and a diagnosis of unilateral PCV or typical exudative AMD or ERM. Patients were excluded if they had an unknown cause of choroidal neovascularization or a cause other than age, such as myopia, angioid streaks, trauma, or uveitis. Other exclusion criteria were as follows: diabetic retinopathy more severe than moderate nonproliferative diabetic retinopathy, central serous chorioretinopathy (CSC), optic atrophy, pathologic myopia, macular hole, retinal vascular disease, uveitis, cataract surgery within the previous three months, or any history of previous vitreoretinal surgery.

A diagnosis of PCV was concluded if sub-retinal reddish-orange nodules were visible on fundus photography with polypoidal structures accompanied with a branching vascular network on ICGA [17]. Typical exudative AMD was diagnosed when serous or hemorrhagic exudative change was present with accompanying drusen or pigmentary changes on fundus photography, with choroidal neovascularization confirmed on fluorescein angiography or ICGA without findings of PCV or retinal angiomatous proliferation. ERM was diagnosed when cellophane macular reflex or premacular fibrosis was identified on fundoscopy with a hyper reflective membrane confirmed on OCT.

Fundus photography and spectral domain OCT images of the uninvolved fellow eyes of these patients were collected and analyzed. In Groups 1 and 2, the ICGA images of the contralateral eyes were also analyzed. The eyes were analyzed by two independent masked observers who were blinded to the diagnosis of the contralateral eye. In cases with disagreement between the two observers, a third independent blinded observer analyzed the images, and the final analysis was decided based on the majority rule.

### Evaluation of color fundus photographs

**Fig. 1:**
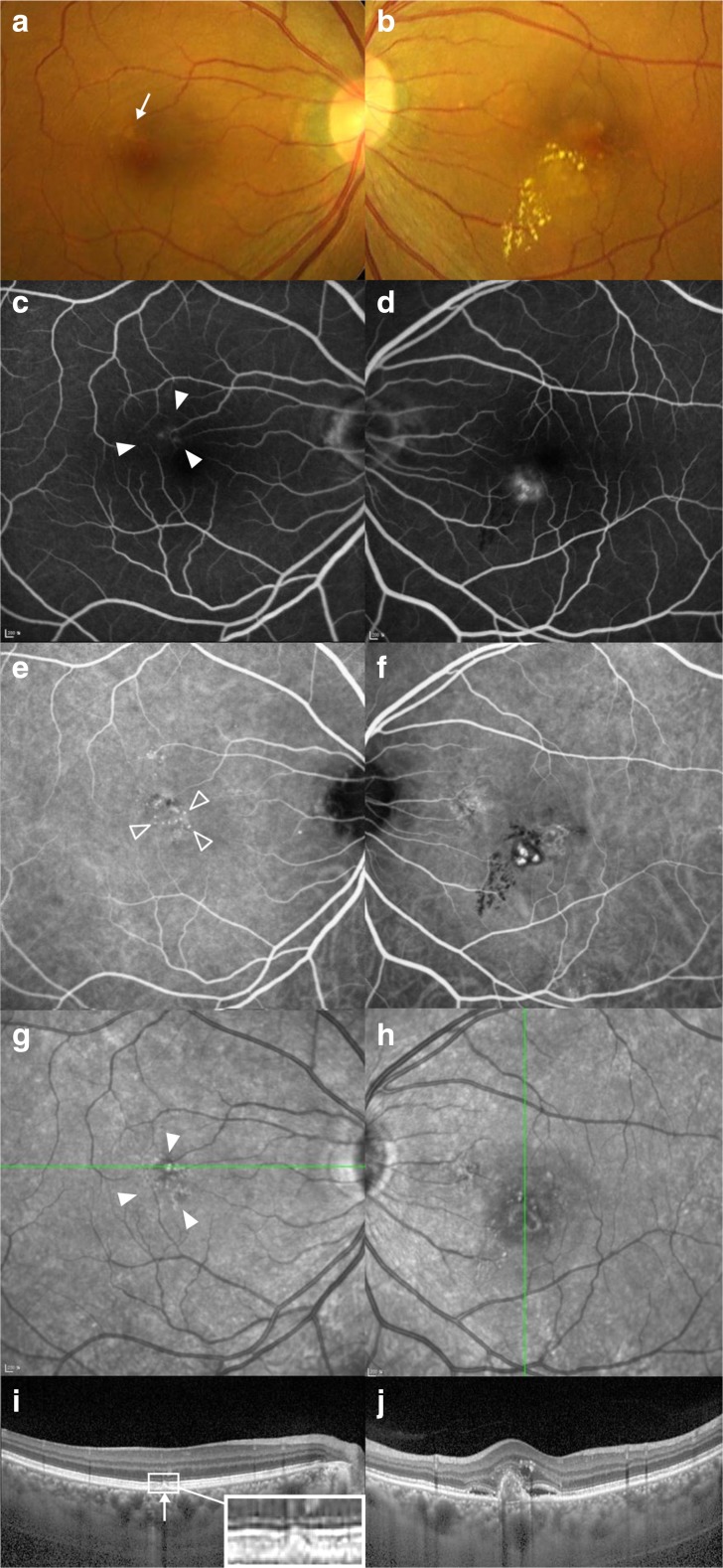
Fundus color photographs (**a, b**), fluorescein (**c, d**) and indocyanine green (**e, f**) angiographs, infrared images (**g, h**) and optical coherence tomographs (**i, j**) of a 58-year-old man with unilateral polypoidal choroidal vasculopathy. In the right column, the multimodal images of the left eye with polypoidal choroidal vasculopathy are demonstrated. In the left column, the multimodal images of the uninvolved right eye are demonstrated, which represents the eyes in Group 1. (**a**) Funduscopic examination reveals drusen-like deposits (DLD, arrow) of a grayish yellow-colored sub-retinal deposit with irregular but discrete margins in the parafoveal area. (**c, g**) Pigmentary changes (*solid arrow heads*) adjacent to DLDs visualized by fluorescein angiography and fundus autofluorescence imaging. (**e**) Mild choroidal hyperpermeability and punctate hyperfluorescent spots (*open arrow heads*) on indocyanine green angiography. In this case, a DLD is spatially correlated with punctate hyperfluorescent spots (**i**) Optical coherence tomography scanning over the DLD reveals subretinal deposits, different from that of soft drusen, which usually show dome-like elevation due to sub-retinal-pigment-epithelial accumulation. The subfoveal choroidal thickness is 256 μm

**Fig. 2:**
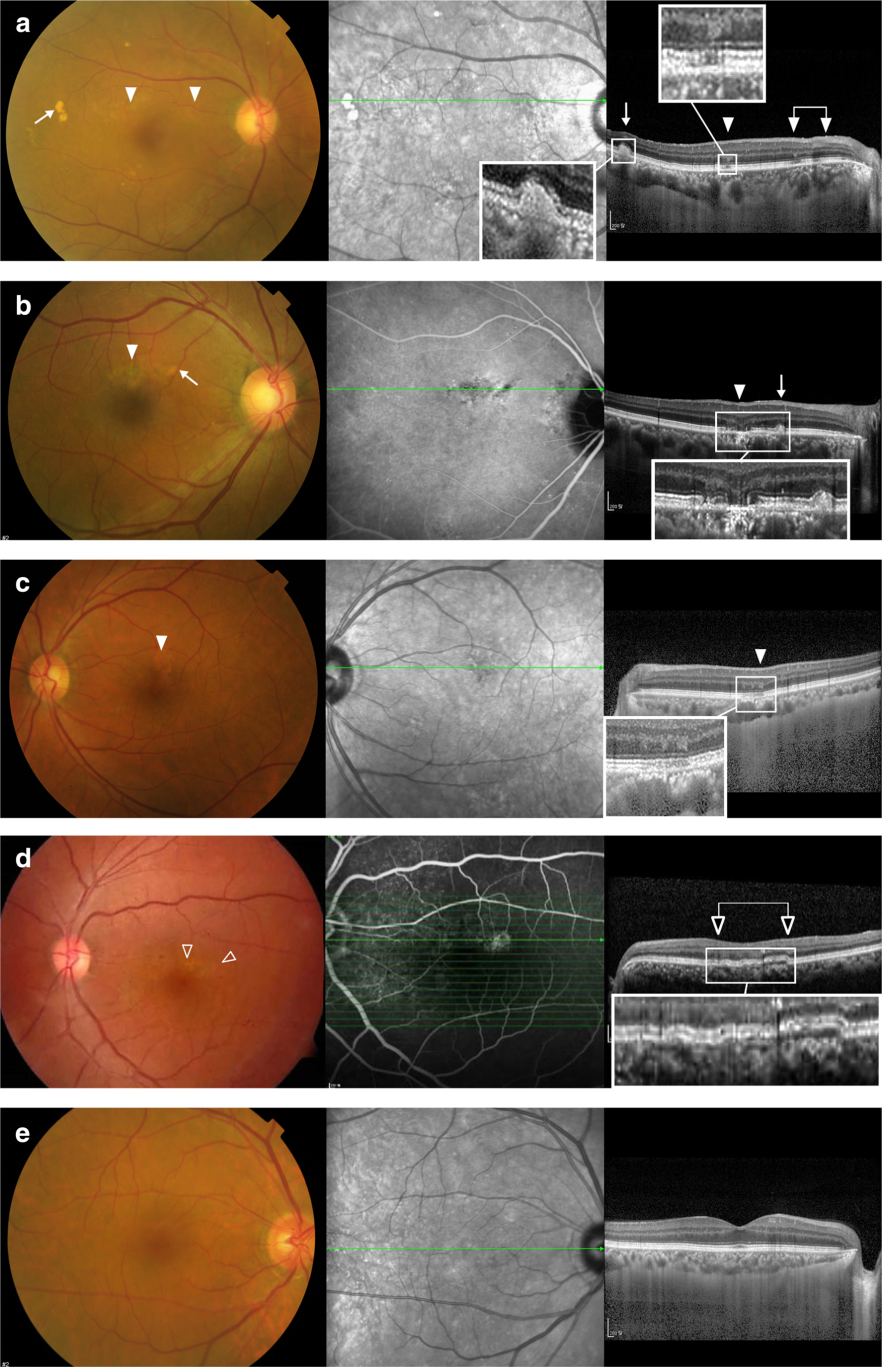
Funduscopic and optical coherence tomographic (OCT) images in the unaffected fellow eyes of patients with unilateral polypoidal choroidal vasculopathy are presented. All cases are from Group 1. (**a, b**) These eyes show drusen-like deposits (DLD, *arrows*) and pigmentary changes (*solid arrow heads*). There are DLDs represented by yellowish deposits with irregular but discrete margins. The DLD manifests as an amorphous subretinal deposit usually disrupting the ellipsoid zone on OCT. OCT manifestations of pigmentary changes range from mild attenuation in the interdigitation zone (**a**) to severe disruption in the outer retina involving the ellipsoid zone and even the external limiting membrane (**b**). Choroidal thickening is remarkable in both cases. (**c, d**) These eyes show only pigmentary changes on funduscopy. The third row case exhibits mild disruption in the interdigitation zone and retinal pigment epithelium/Bruch’s complex on OCT. In contrast, a double layer sign (*open arrow heads*) on OCT is conspicuous in the fourth row case. (**e**) In a significant proportion of eyes in Group 1, no specific abnormality was noted, as in this case

In fundus photographs, drusen-like deposits (DLD), drusen, and pigmentary change within 30 to 35 degrees of the fundus were evaluated. DLD were defined as sub-retinal or sub-retinal-pigment-epithelial grayish yellow deposits larger than 63 μm in size with irregular but discrete margins (Figs. 1a and 2a, b). The number of patients with DLD in each group was evaluated, and the proportion of patients with DLD of 125 μm or larger size was also evaluated in each group. Drusen were further grouped into small hard drusen, soft drusen, and reticular pseudodrusen. Small hard drusen were defined as drusen smaller than 63 μm, and soft drusen were defined as drusen that were 63 μm or larger with round and soft borders. Reticular pseudodrusen were defined as drusenoid deposits with a yellowish interlacing network on fundus photography, hypofluorescence on infrared reflectance images, and evidence of corresponding sub-retinal deposits on OCT [18–21]. Pigmentary change was defined as depigmentation or reactive hyperpigmentation with a size larger than 1/8 disc diameter on fundus photography (Fig. 2b, c, d). The proportions of eyes with these findings were evaluated in each group.

### Evaluation of spectral domain optical coherence tomography images

The presence of a double layer sign, elevation by DLD or soft drusen, elevation by pigment epithelial detachment, and disruption of the retinal pigment epithelial layer due to pigmentary change were evaluated on OCT. A double layer sign was defined as two highly reflective layers visible at the retinal pigment epithelium (Fig. 2d) [22, 23]. Elevation by DLD or soft drusen was defined as elevation over the nearby interdigitation zone line, formerly called the cone outer segment tip line [24], on OCT in the area of DLD or soft drusen. The criterion for defining elevation by pigment epithelial detachment was elevation over the nearby interdigitation zone line by serous pigment epithelial detachment. Disruption of the retinal pigment epithelial layer due to pigmentary change was defined as retinal pigment epithelial layer disruption on the OCT scan of the area of pigmentary change on fundus photography.

### Evaluation of indocyanine green angiography images

In Groups 1 and 2, the presence of choroidal hyperpermeability, punctate hyperfluorescence spots, and late geographic hyperfluorescence on ICGA were evaluated in all eyes. The spatial correlation of punctate hyperfluorescence spots and choroidal hyperpermeability or DLD was evaluated. Choroidal hyperpermeability was defined as multifocal hyperfluorescent areas with blurred margins after a gradual increase in choroidal hyperfluorescence intensity 10 minutes after infusion of indocyanine green [9]. Punctate hyperfluorescence spots were defined as punctate hyperfluorescence in the mid- to late phase of ICGA (Fig. 1c) [25]. The number of punctate hyperfluorescence spots was grouped into 2 or less, 3 to 10, 11 to 20, 21 to 40, or more than 40. Late geographic hyperfluorescence was defined as hyperfluorescent lesions with a clearly demarcated geographic margin about 10 minutes after injection of indocyanine green dye [26].

### Statistical analysis

Statistical analysis was performed using SPSS software version 18.0 (SPSS, Inc., Chicago, IL, USA). To identify whether there was any difference between the three groups, we conducted analysis of variance (ANOVA) for continuous variables and chi-square test for non-continuous variables. P value less than 0.05 is considered significant.

If the above analysis confirmed any significant difference, a comparison between two groups was then conducted by independent Student’s T-test for continuous variables and Fisher’s exact test for non-continuous variables. A P value less than 0.016 after Bonferroni correction was considered significant.

## Results

The study included 154 eyes of 154 unilateral PCV patients in Group 1, 59 eyes of 59 unilateral typical exudative AMD patients in Group 2, and 63 eyes of 63 unilateral ERM patients in Group 3. The mean age was 67.2 ± 8.1 years in Group 1, 71.6 ± 8.9 years in Group 2, and 66.9 ± 9.4 years in Group 3 (Table 1). There was no statistical difference in age between Group 1 and Group 3, which was the age-matched control group (*p* = 0.182). There were significantly more male patients in Group 1 compared to Groups 2 and 3 (*p* = 0.005 and *p* < 0.001, respectively) (Table 1).

**Table 1:** Basic demographic characteristics of the study subjects

	Group 1	Group 2	Group 3	Overall *P* value^*^	*P* value^†^	*P* value^‡^	*P* value^**^
N (eyes)	154	59	63				
Age (years)	67.2 ± 8.1	71.6 ± 8.9	66.9 ± 9.4	0.002	0.182	0.006	0.001
M : F	2.2:1	0.9:1	0.7:1	<0.001	<0.001	0.465	0.005

Group 1: fellow eye of unilateral polypoidal choroidal vasculopathy; Group 2: fellow eye of unilateral typical exudative age-related macular degeneration; Group 3: fellow eye of unilateral epiretinal membrane, as a control

* comparison of all Groups, by ANOVA for continuous variables and chi-square test for non-continuous variables. P value less than 0.05 is considered significant

The independent Student’s T-test was used for the comparison of age, and Fisher’s exact test was used for the comparison of gender in each group. P value less than 0.016 after Bonferroni correction is considered significant

^†^ comparison of Groups 1 and 3

^‡^ comparison of Groups 2 and 3

^**^ comparison of Groups 1 and 2

### Fundus photographic findings

On analysis of the frequency of drusen in each group, soft drusen and reticular pseudodrusen were both more common in Group 2 than Groups 1 and 3 (*p* < 0.001) (Table 2). The frequency of small hard drusen was not different between groups (*p* = 0.09). DLDs were found in the highest frequency in Group 1 (24.7 %), which was significantly higher than in Groups 2 and 3 (*p* < 0.001 and *p* = 0.015, respectively). Also, DLDs of 125 μm or larger were found in 19.5, 3.4, and 3.2 % of patients in Groups 1 through 3, respectively, with a significantly larger percentage found in Group1 (*p* = 0.002 and *p* = 0.001, respectively) (Table 2). The DLDs were usually heterogeneous in size within a given eye, and their distribution was usually not central (Figs. 1a and 2a, b). There was no significant difference in presence of DLDs between Groups 2 and 3 (*p* = 0.275). The frequency of pigmentary change was 39.6, 35.6, and 14.3 % in Groups 1 through 3, respectively. Group 3 showed significantly less pigmentary change than the other two groups (*p* < 0.001 and *p* = 0.011, respectively).

**Table 2:** Frequency of funduscopic findings in each group

	Group 1	Group 2	Group 3	Overall *P* value*	*P* value^†^	*P* value^‡^	*P* value^**^
N. of participants	154	59	63				
Drusen type							
Small hard drusen	33.8 %	27.1 %	19.0 %	0.091			
RPD	0.6 %	22.0 %	1.6 %	<0.001	0.497	<0.001	<0.001
Soft drusen	4.5 %	25.4 %	8.0 %	<0.001	0.110	<0.001	<0.001
Presence of DLD	24.7 %	3.4 %	9.5 %	<0.001	0.015	0.275	<0.001
Size of DLD				<0.001	0.001	1.000	0.002
<125 μm	80.5 %	96.6 %	96.8 %				
> 125 μm	19.5 %	3.4 %	3.2 %				
Pigmentary changes	39.6 %	35.6 %	14.3 %	0.001	<0.001	0.011	0.639

Group 1: fellow eye of unilateral polypoidal choroidal vasculopathy; Group 2: fellow eye of unilateral typical exudative age-related macular degeneration; Group 3: fellow eye of unilateral epiretinal membrane, as a control

Drusen-like deposits (DLD) were defined as sub-retinal or sub-retinal pigment epithelial grayish yellow deposits larger than 63 μm in size with irregular but discrete margins

RPD: reticular pseudodrusen

* comparison of all Groups; P value less than 0.05 is considered significant

^†^ comparison of Groups 1 and 3; P value less than 0.016 is considered significant

^‡^ comparison of Groups 2 and 3; P value less than 0.016 is considered significant

^**^ comparison of Groups 1 and 2; P value less than 0.016 is considered significant

### Optical coherence tomography findings

A double layer sign was found in 9.1, 10.2, and 0.0 % of eyes in Groups 1, Group 2, and Group 3, respectively, and was significantly less frequent in Group 3 than Groups 1 and 2 (*p* = 0.012 and *p* = 0.011, respectively) (Table 3). Elevation by DLD or soft drusen was found in 16.9, 37.3, and 1.6 % of eyes from each group, respectively, and was significantly less frequent in Group 3 than Groups 1 and 2 (*p* = 0.001 and *p* < 0.001, respectively). Elevation due to DLD was usually accompanied by disruption of the ellipsoid zone in the corresponding area, and there was a certain degree of hyper reflectivity in the inner borders of DLDs. In contrast to soft drusen, the DLDs usually manifests as asymmetric or triangular-shaped subretinal deposits on OCT (Fig. 2a, b). Elevation by pigment epithelial detachment was found in 5.8, 8.5, and 0 % of each group, respectively, and was significantly more common in Group 2 compared to Group 3 (*p* = 0.024). Disruption of the retinal pigment epithelial layer due to pigmentary change was found in 4.5, 8.5, and 1.6 %, respectively, with no significant difference among the groups.

**Table 3:** Frequency of spectral domain optical coherence tomography findings in each group

	Group 1	Group 2	Group 3	Overall *P* value*	*P* value^†^	*P* value^‡^	*P* value^**^
N. of participants	154	59	63				
Double layer sign	9.1 %	10.2 %	0.0 %	0.015	0.012	0.011	0.797
Elevation by DLD or soft drusen	16.9 %	37.3 %	1.6 %	<0.001	0.001	<0.001	0.003
Elevation by PED	5.8 %	8.5 %	0.0 %	0.049	0.062	0.024	0.539
Disruption of RPE	4.5 %	8.5 %	1.6 %	0.203			
Choroidal thickness, μm	261.4 ± 99.0	178.2 ± 101.0	207.4 ± 77.6	<0.001	<0.001	0.080	<0.001

Group 1: fellow eye of unilateral polypoidal choroidal vasculopathy; Group 2: fellow eye of unilateral typical exudative age-related

macular degeneration; Group 3: fellow eye of unilateral epiretinal membrane, as a control

DLD, drusen-like deposits; PED, pigment epithelial detachment; RPE, retinal pigment epithelium

* comparison of all Groups; P value less than 0.05 is considered significant

^†^ comparison of Groups 1 and 3; P value less than 0.016 is considered significant

^‡^ comparison of Groups 2 and 3; P value less than 0.016 is considered significant

^**^ comparison of Groups 1 and 2; P value less than 0.016 is considered significant

The mean subfoveal choroidal thicknesses in the eyes of Groups 1 to 3 was 261.4 ± 99.0, 178.2 ± 101.0, and 207.4 ± 77.6 μm, respectively. The thickness in Group 1 was significantly greater than those of Groups 2 and 3 (*p* < 0.001, < 0.001, respectively). The thickness of Group 2 tended to be less than that of Group 3, although this difference was not statistically significant (*p* = 0.080) (Table 3).

### Indocyanine green angiography findings

On comparison of ICGA findings of Group 1 and 2, choroidal hyperpermeability was found in 23.4 and 6.8 % of eyes, respectively, and was significantly more frequent in Group 1 compared to Group 2 (*p* = 0.005) (Table 4). The number of punctate hyperfluorescence spots was not different among the two groups (*p* = 0.647). Late geographic hyperfluorescence was found in 9.7 and 1.7 % of eyes in Groups 1 and 2, respectively (*p* = 0.076). Spatially correlated DLDs and punctate hyperfluorescence spots were found in 18.2 % of eyes in Group 1, and 0 % of Group 2 (*p* < 0.001) (Fig. 1e). Spatially correlated punctate hyperfluorescence spots and choroidal hyperpermeability were found in 8.4 % of eyes in Group 1 and 5.1 % of Group 2 (*p* = 0.565).

**Table 4:** Indocyanine green angiography findings in Groups 1 and 2

	Group 1	Group 2	*P* value^†^
N. of participants	154	59	
Choroidal hyperpermeability	23.4 %	6.8 %	0.005
Punctate hyperfluorescence spot			
0–2	23.4 %	28.8 %	0.647
3–10	20.8 %	23.7 %	
10–20	27.3 %	18.6 %	
20–40	19.5 %	16.9 %	
>40	9.1 %	11.9 %	
Late geographic hyperfluorescence	9.7 %	1.7 %	0.076
Spatially correlated punctate hyperfluorescence spots and DLD	18.2 %	0.0 %	<0.001
Spatially correlated punctate hyperfluorescence spots and choroidal hyperpermeability	8.4 %	5.1 %	0.565

Group 1: fellow eye of unilateral polypoidal choroidal vasculopathy; Group 2: fellow eye of unilateral typical exudative

age-related macular degeneration; Group 3: fellow eye of unilateral epiretinal membrane, as a control

DLD, drusen-like deposits

^†^ comparison of Groups 1 and 2

## Discussion

In this study, the uninvolved fellow eyes of active PCV or typical exudative AMD patients were studied. The bilateral PCV has been reported in about 15 % of cases [4, 7, 15, 27], and the probability of exudation in the fellow eye in neovascular AMD is at least 13 % [28]. The probability of the fellow eye developing the same disease in unilateral cases of active exudative maculopathy is higher than 1.2 % [29], which is the incidence in the general population. Previous reports also indicate that the diagnosis in the fellow eyes, if involved, is almost always the same subtype of neovascular AMD [4, 15]. Therefore, studying changes in the uninvolved fellow eye without active exudative maculopathy might be an effective method of evaluating precursor lesions of these diseases.

One of the major findings of this study was that soft drusen and reticular pseudodrusen were more common in Group 2, the typical exudative AMD group, than the normal control Group 3. This supports the previously well-known finding that soft drusen and reticular pseudodrusen are early precursor lesions of typical exudative AMD, and further supports the validity of this study method. Soft drusen and reticular pseudodrusen were found significantly more often in Group 2 than in Group 1, the PCV group, while there was no significant difference between Groups 1 and 3. Therefore, soft drusen and reticular pseudodrusen are unlikely to be precursor lesions of PCV. This might also serve as evidence that PCV has a different pathophysiologic mechanism from typical exudative AMD. DLD, especially those larger than 125 μm, were found significantly more often in the PCV group than the control group or exudative AMD group. Also, the frequency of DLD was not different between the typical exudative AMD group and the control group. In other words, soft drusen and reticular pseudodrusen can be thought of as early precursor lesions heralding the development of late exudative AMD, while DLD (especially large lesions) can be thought of as the early precursor lesion of PCV.

**Fig. 3:**
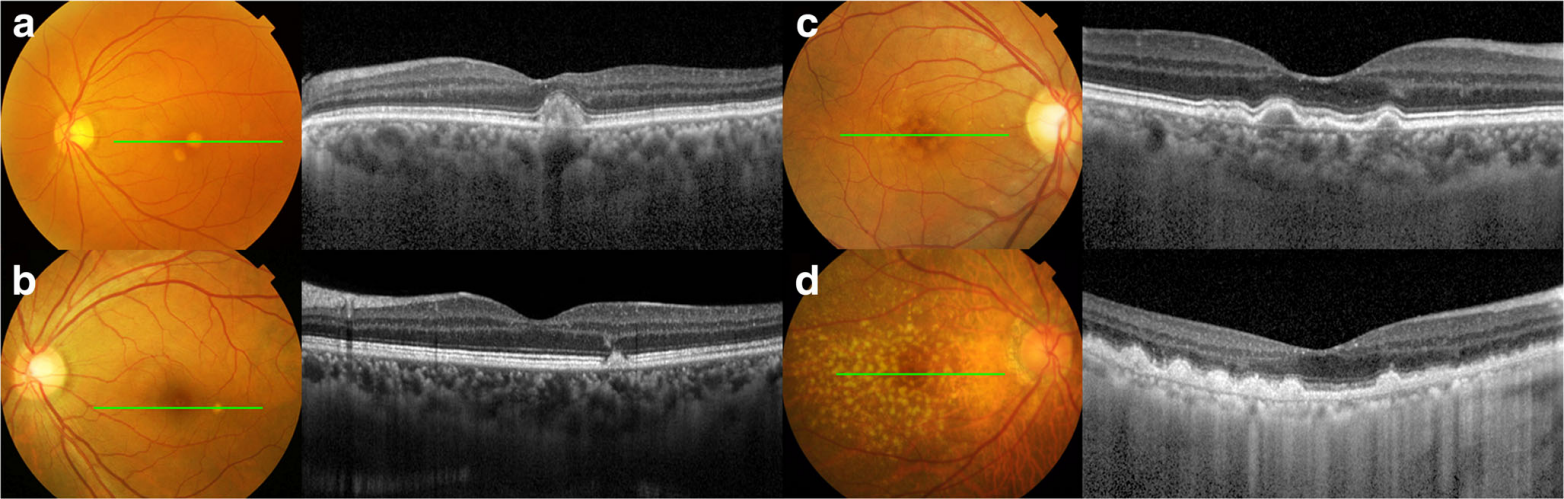
Funduscopic and optical coherence tomographic (OCT) images comparing drusen-like deposits (DLD, **a**, **b**) and soft drusen (**c**, **d**). Compared with soft drusen, DLD exhibits a relatively larger size with a non-circular and irregular margin. Asymmetric distribution between two eyes also characterizes DLD. DLD manifests as an amorphous subretinal deposit usually disrupting the ellipsoid zone on OCT

In this study, DLD was defined as a sub-retinal or sub-retinal-pigment-epithelial grayish yellow deposit of 63 μm or larger in size with irregular but discrete margins. The largest difference between DLD and typical drusen on funduscopy is the irregularity of the margin, which might result from clustering of smaller deposits at the margins of previous lesions (Fig. 3). Compared with true drusen, DLD exhibits a relatively large size with a non-circular and irregular margin and often accompanies pigmentary changes.

After reporting a case series of pachychoroid pigment epitheliopathy that manifested drusen-like lesions and a thick choroid [30], Freund and associates have proposed the existence of a pachychoroid pigment epitheliopathy—pachychoroid neovasculopathy—PCV axis [31]. DLD observed in the present study are possibly the same lesions as they termed drusen-like lesions. The present results of Group 1 eyes showing DLD, choroidal hyperpermeability, and choroidal thickening further support their hypothesis. DLDs are accompanied by pigmentary changes and yellowish sub-retinal precipitates that are composed of macrophages and outer segment photoreceptor shedding [32, 33]. The initially dot-like precipitates become confluent and increase in size, and the irregular margins of DLDs observed in the current study might be produced by the confluence of these dot-like precipitates. Also, in Group 1, the spatial correlation of punctate hyperfluorescence spots and DLDs was confirmed, further supporting this developmental mechanism of DLD.

A double layer sign, elevation due to DLD or soft drusen, and disruption of the retinal pigment epithelial layer due to pigmentary change were found in significantly higher frequencies on OCT in Group 1 than in the control Group. In contrast to soft drusen, the DLDs usually manifest as asymmetric or triangular-shaped subretinal deposits with a certain degree of hyper reflectivity on OCT. Because sub-retinal or sub-retinal-pigment-epithelial deposits including DLD were associated with macular diseases other than AMD, it can be difficult to differentiate funduscopically from true drusen, OCT is useful for differentiating DLD and drusen and for evaluating choroidal thickening. In previous reports, drusen were not an uncommon finding in PCV [4, 34]. It is possible that a significant proportion of drusen described in previous studies of PCV might have been DLD. As OCT is useful for differentiating DLD and true drusen, it might contribute to identifying the precursor lesions of PCV, especially when combined with fundus autofluorescence imaging.

Identifying the precursor lesion of PCV has various implications. First, the pathophysiological process can be identified and addressed before the development of active PCV via systemic or genomic studies. Second, proper disease staging, instruction, and surveillance of early lesions will be possible. Third, treatment methods to delay or prevent progression might be developed through further studies of high-risk groups. Based on the results of this study, a nation-wide cohort study to identify the real risk of developing active PCV in subjects with DLDs and pigmentary changes is currently being launched.

This study has several limitations. First, patients with PCV were recruited from seven referral hospitals to secure a sufficient number of unequivocal cases. Eyes with typical exudative AMD or ERM were enrolled from a reading center. Thus, the numbers in Group 1 and Group 2 do not reflect the actual prevalence of exudative AMD subtypes in Asia [4, 5]. However, we did not expect this to significantly affect the main results of the current study. Second, this study was a multi-center, cross-sectional study in which heterogeneous equipment for angiography and spectral domain OCT was involved. Third, some OCT scans were limited in use for evaluating DLD or pigmentary changes, and this might have affected the results. Despite these limitations, this study is significant because it included a relatively large number of uninvolved fellow eyes of unilateral PCV cases, and, as far as the authors are aware, this is the first study to suggest DLD as the precursor lesion of PCV.

In conclusion, this study suggests that pigmentary change and sub-retinal or sub-retinal-pigment-epithelial grayish yellow deposits with an irregular margin (DLD) are early precursor lesions of PCV. In particular, DLDs larger than 125 μm in size are specific precursor lesions of PCV. These lesions may have been formed from previous subclinical or chronic inactive central serous chorioretinopathy. Further large-scale longitudinal studies are needed to confirm this conclusion.

## Appendix

^1^Department of Ophthalmology, Samsung Medical Center, Sungkyunkwan University School of Medicine, Seoul, Korea.

^2^Department of Ophthalmology, the Institute of Vision Research, Yonsei University College of Medicine, Seoul, Korea.

^3^Department of Ophthalmology, Seoul National University Bundang Hospital, Seoul National University College of Medicine, Kyeounggi, Korea.

^4^Department of Ophthalmology, Kyungpook National University Hospital, Daegu, Korea.

^5^Department of Ophthalmology, KyungHee University, Seoul, Korea.

^6^Department of Ophthalmology, Korea University College of Medicine, Seoul, Korea.

^7^Department of Ophthalmology, Chungnam National University Hospital, Daejeon, Korea.

^8^Department of Ophthalmology, Busan Paik Hospital, Inje University, Pusan, Korea.
